# Case Report: Intracranial Hypertension Secondary to Guillain-Barre Syndrome

**DOI:** 10.3389/fped.2020.608695

**Published:** 2021-01-20

**Authors:** Christina Doxaki, Eleftheria Papadopoulou, Iliana Maniadaki, Nikolaos G. Tsakalis, Konstantinos Palikaras, Pelagia Vorgia

**Affiliations:** ^1^Department of Pediatrics, University General Hospital of Heraklion, Crete, Greece; ^2^Department of Ophthalmology, University General Hospital of Heraklion, Crete, Greece; ^3^Institute of Molecular Biology and Biotechnology (IMBB), Foundation for Research and Technology - Hellas (FORTH), Crete, Greece

**Keywords:** intracranial hypertension, Guillain–Barre syndrome, hydrocephalus, papilledema, headache

## Abstract

Guillain-Barre Syndrome (GBS), a common cause of acute flaccid paralysis, is characterized by a rapidly progressive, usually symmetric weakness of the extremities. Headache and intracranial hypertension (ICHT) are very rare complications of GBS. Herein we report our current case of an obese girl with typical signs of GBS associated with autonomic dysfunction, cranial nerve deficits and increased intracranial pressure (ICP). We also perform a systematic study presenting and discussing previous case reports of GBS associated with ICHT, papilledema or hydrocephalus, highlighting the differences of the current case compared to previous studies. Although intracranial hypertension is a rare complication of pediatric GBS, clinicians should promptly detect it. Obesity may be a predisposing factor, given the strong association between idiopathic intracranial hypertension (IIH) and weight gain. Neurological evaluation, fundus examination and low threshold for intracranial imaging should be an integral part of medical practice in case of obesity, headache or visual changes in GBS patients.

## Introduction

Guillain–Barre syndrome (GBS), a potentially life-threatening postinfectious condition is an acute immune-mediated polyradiculoneuropathy characterized by a rapidly progressive, usually bilateral weakness of the limbs, hypo- or areflexia, often accompanied by sensory symptoms, cranial nerve and autonomic dysfunction (1–4). Molecular mimicry and cross-reactive immune response play a crucial role in its pathogenesis. Intravenous immunoglobulin (IVIg) and plasma exchange are effective treatments in GBS. Although medical treatment can improve or stabilize patients, subsequent deterioration may happen after initial improvement, suggesting a severe disease with slow recovery phase and poor prognosis. Among the uncommon symptoms and complications of GBS are headache, papilledema and intracranial hypertension (5–9).

In this study, we describe a case of an obese girl presenting symptoms of intracranial hypertension (ICHT) secondary to GBS. A systematic review of previous case reports associated with GBS and intracranial pressure (ICP) is included, with the aim to highlight this rare clinical manifestation of GBS in children and analyse the differences of this case compared to previous studies (Table 1). Furthermore, clinicians should be alerted for punctual diagnosis and repeated ophthalmologic reassessments in order to exclude ICHT upon headache and/or visual changes in GBS setting.

**Table 1:** Intracranial hypertension in GBS.

**Author**	**Age (years)/sex (female/male)**	**CSF pressure (mmH2O)**	**CSF protein (mg/dL)**	**ICP signs Diplopia/headache/papilloedema/hydrocephalus before/after GBS signs**	**VP shunt**	**IVIg /PLEX**	**Drug therapy (acetazolamide/ corticosteroids)**	**Recover time (mo)**
Joynt (10)	11 (M) obese	500	480	Diplopia and papilledema 2 weeks after limb weakness	-	-	-	NM
Gilmartin and Chien (11)	1 mo (F)	245	230 → 870	Seizures and hydrocephalus 20 days after limb paralysis	Yes	No	No	2
Reid and Draper (12)	16 (M)	220 → 330	160 → 360	Nausea, vomiting, headache, papilledema, and hydrocephalus 10 weeks after limb weakness	No	PLEX	No	5
Farrell et al. (13)	8 (F)	250	230 → 507	Papilledema and hydrocephalus 1 mo after limb weakness	No	No	No	6
Hantson et al. (14)	16 (M)	NM	640	Seizures and hydrocephalus wo papilledema 2 mo after limb weakness	No	PLEX	No	6
Ersahin et al. (15)	10 (M)	150	246	Headache and diplopia (papilledema + hydrocephalus) 11 weeks after limb weakness	Yes	No	No	10
Mewasingh et al. (16)	2 (F)	200	Normal	Ataxia, vomits, strabismus 2 weeks before Miller-Fisher signs	No	IVIg	Corticosteroids	3
Mewasingh et al. (16)	9 (F)	300	Normal	Headache, diplopia, nausea 5 days before Miller-Fisher signs	No	IVIg	Acetalozamide	1
Barzegar et al. (7)	21mo (F)	(>200)	350	Seizures and hydrocephalus 15 days after limb weakness	No	IVIg	Corticosteroids	~12
Incesik et al. (17)	14 (M)	180	78	Visual loss and bilateral papillitis 3 days prior to weakness–AMSAN form)	No	IVIg	Corticosteroids	2
Zhao et al. (18)	14 (F)	190 → 290	112 → 117	Papilledema and hydrocephalus 1.5 mo after limb weakness and areflexia	No	IVIg X2	No	7
Present Case	12 (F) obese	460	245	ICP (+ papilloedema) 3 weeks after limb weakness	No	IVIg	Both	1
Morley et al. (19)	46 (F)	260-280	200 → 400	Headache and papilledema 2 weeks after limb weakness	No	No	Prednisone	3
Morley et al. (19)	24 (F)	180-235	140 → 950	Headaches, neck stiffness, vomiting, papilledema 10 days after limb weakness (a relapse of GBS after 5 mo)	No	No	Prednisone	12
Morley et al. (19)	59 (M)	160	180	Headache and papilledema 2 weeks before limb weakness	No	No	No	12
Morley et al. (19)	23 (M)	75 → 170	1800 → 300	Papilledema 3 weeks after limb weakness	No	No	Prednisone	6
Janeway and Kelly (20)	21 (M) overweight	200 → 480	205 → 1250	Headache, diplopia, papilledema, and hydrocephalus 2 mo after limb weakness	Yes	No	Coricotropin	12
Sullivan et al. (21)	24 (F) obese	310	Normal → 230	Headache, nausea, vomiting, papilledema 5 days before limb weakness	No	No	Prednisone	6
Ropper and Marmarou (22)	27 (M)	NM → 615	727	Headache, papilledema wo hydrocephalus 3 weeks after limb weakness	No	PLEX	Prednisone	NM
Weiss et al. (23)	22 (F)	600	Normal → 106	Headache, blurred/double vision, papilledema wo hydrocephalus 5 days before limb weakness.	No	NM	NM	NM
Kharbanda et al. (24)	35 (F)	420	20	Headache, diplopia, visual loss 2 weeks before GBS onset	No	No	Both	NM
Pyati et al. (25)	26 (F)	300 → 420	72 → 540	Headache, papilledema wo hydrocephalus at the onset of GBS	No	PLEX	Prednisone	~ 3
Liu et al. (26)	67 (M)	145	146	Chronic hydrocephalus wo headache or papilledema, prior to GBS onset NPH: normal pressure hydrocephalus	Yes	Both	No	12
Ozdemir et al. (27)	32 (M)	300	180	Headache, nausea, vomiting and hydrocephalus 5 days before GBS onset	Yes	Both	No	12
Alrohimi and Jassal (28)	33 (F) obese	143	500	Headache and vision changes, ICP, wo hydrocephalus 2 weeks after limb weakness	Yes	IVIg	Acetazolamide	12
Wen (29)	43 (F)	NM	86	Blurred vision, diplopia 5 days before limb weakness	No	IVIg	Dexamethasone	~1
Wang et al. (3)	26 (F)	280 → 400	179 → 190	Headache, diplopia, ICP simultaneously with limb weakness and relapse 1 mo later with visual loss and hydrocephalus	Yes	IVIg	Corticosteroids	2

*This table shows previous case reports with intracranial hypertension in the setting of GBS*.

*ICP, intracranial pressure; PLEX, Plasma Exchange, VP shunt: ventriculo-peritoneal shunt*.

*NM refers to non-mentioned*.

## Case Report

**Figure 1:**
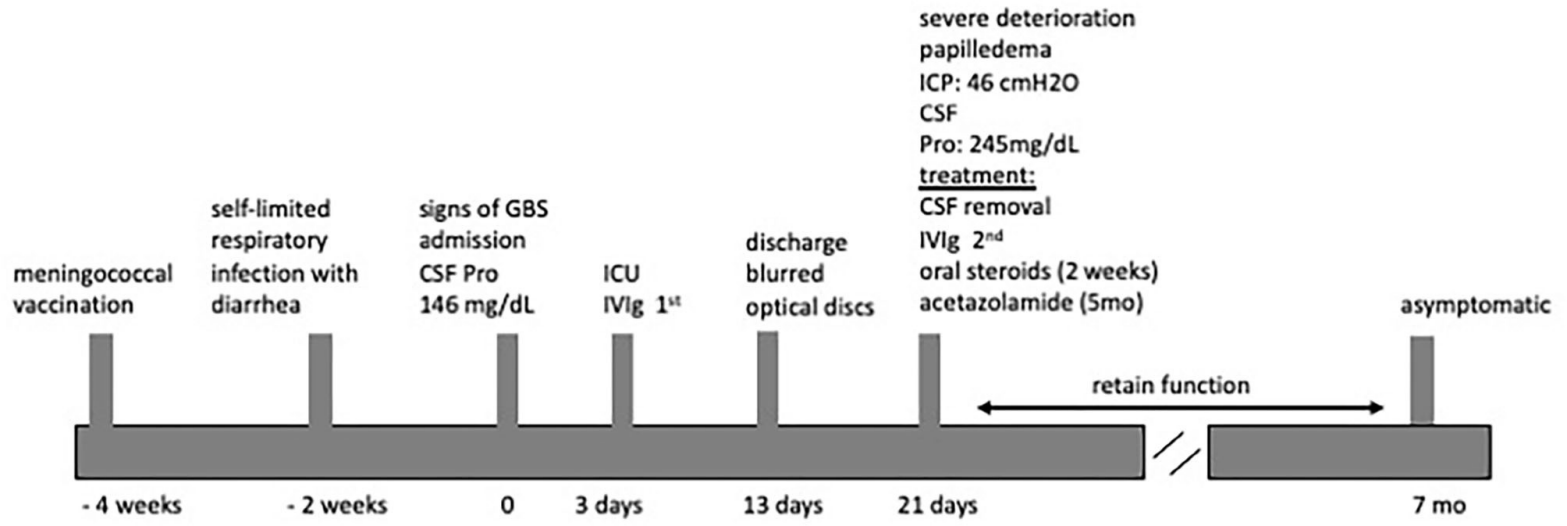
Timeline of present case report.

We describe the case of a fully immunized, obese 12-years-old girl (BW 68 kg, BMI 32.3), who was admitted to our hospital in 2018 with a 2-days progressive ascending weakness and aching in both legs, which had slightly been spread to her arms. An acute self-limited respiratory infection with diarrhea and a meningococcal vaccination, 2 and 4 weeks earlier respectively, were preceded the onset of weakness (Figure 1).

**Figure 2:**
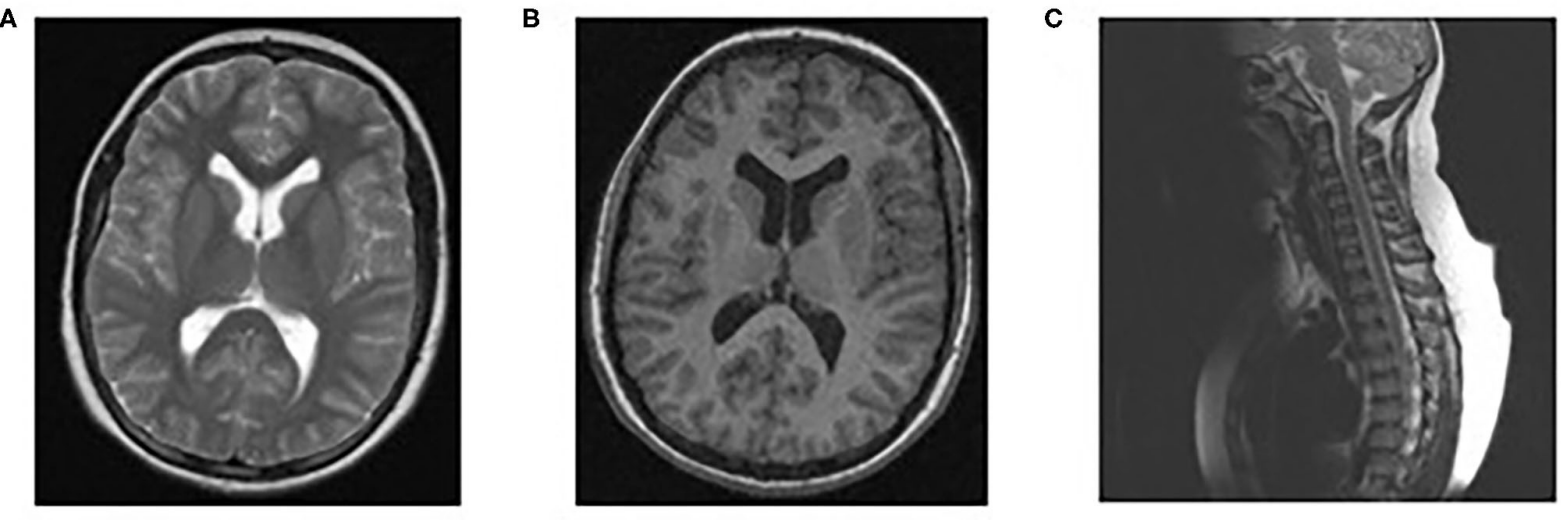
Normal findings of brain/spinal MRI. MRI brain scan T1 **(B)** and T2 **(A)**, MRI spine sagittal **(C)**.

Physical examination revealed paraparesis, areflexia, and weakness restricted to her legs. However, over the course of her illness moderate arm weakness was observed. Deep tendon reflexes were absent while sensory examination was normal. Muscle strength was 3/5 proximally and 1-2/5 distally of all limbs using the Medical Research Council scale (30). Cranial nerve examination revealed bilateral facial weakness and incomplete eyelid closure while bilateral fundoscopy had normal findings. Brain/spine MRI was unremarkable (Figure 2). CSF analysis revealed cytoalbuminologic dissociation with white blood cell (WBC) of 5 × 10^6^/L, protein at 146 mg/dl and glucose 68 mg/dL (blood glucose 87 mg/dL). On laboratory examination, routine hematological, biochemical, urine, and stool analysis were normal. Serologic tests for cytomegalovirus, herpes simplex virus, Epstein–Barr virus, coxsackie viruses, influenza A and B, enteroviruses and adenovirus, hepatitis A, B, HIV, *Cambylobacter jejuni* and *Mycoplasma pneumoniae* were negative. Thyroid-stimulating hormones and T3/T4, anti-nuclear antibody, anti-tissue and anti-neutrophil cytoplasmic antibodies were normal. Moreover, the antibodies against gangliosides (GM1, GQ1B, GD1B, GT1B, GD1a, GM2, and MAG) were negative. She was immunized for rubella and measles in early childhood (Table 2). Given her vaccination status and virologic investigation of stool samples, we excluded poliovirus as a possible cause. This case was recorded to the National Poliovirus/Enterovirus Reference Laboratory (Hellenic Pasteur Institute), responsible for the investigation of Acute Flaccid Paralysis (AFP). Electromyography (EMG) and nerve conduction studies confirmed the diagnosis of GBS, revealing a severe demyelinating motor polyneuropathy (Table 2).

**Table 2:** Patient's medical information.

Auto-abs	• anti-MAG (-) • anti-GM1,2 (-) • anti-GD a 1b (-) • anti-GQ1b (-)
Anti-nuclear antibodies	anti-ds-DNA(-)
Anti-tissue antibodies	• ASMA (-) • AMA(-) • ANA(-)
Anti-neutrophil cytoplasmic antibodies	• pANCA (-) • cANCA (-)
Rheumatoid Factor	(-)
Laboratory testing	• HIV, HBV, HCV, HAV (-), • CMV IgG (+), IgM (-) • EBV IgG(+), IgM (-) • VZV IgG (+), IgM (-), • Measles IgG (+), IgM (-) • Mumps IgG (-) IgM (-) • HSV1 IgG (+) IgM (-) • HSV2 (-) • ADV IgA (-) • Parvo (-) • Echo(-) • Coxsackie (-) • Mycoplasma pneumoniae (-)
Immunologic blood test	C3 197 mg/dL >187 mg/dL
Stool sample	Normal flora
EMG (electromyography)	“Motor distal demyelinating polyneuropathy of nerves (elevated final latency time) and roots (completely absent or very high F waves latency time, A waves recording) with cranial nerve involvement. Findings compatible with the diagnosis of GBS.”

Due to the rapidly progressing limb weakness and incipient respiratory failure, she was admitted to ICU for monitoring and supportive treatment, 3 days after the onset of her symptoms. In accordance with the diagnosis of GBS, she was directly treated with intravenous immune globulin at the dose of 0.4 g/kg daily for 5 days. Notably, during her hospitalization she also suffered from hypertension (SBP: 145–170 mmHg, DBP: 70–100 mmHg) with mild tachycardia, which was confronted with a selective β1-receptor antagonist.

The patient 10 days after IVIg treatment had a gradual recovery with substantial motor improvement, therefore she was discharged but closely monitored for repeated reassessments, given that 1 day before her discharge, she started complaining about mild headache. Fundoscopy for the first time showed blurred optic discs.

**Figure 3:**
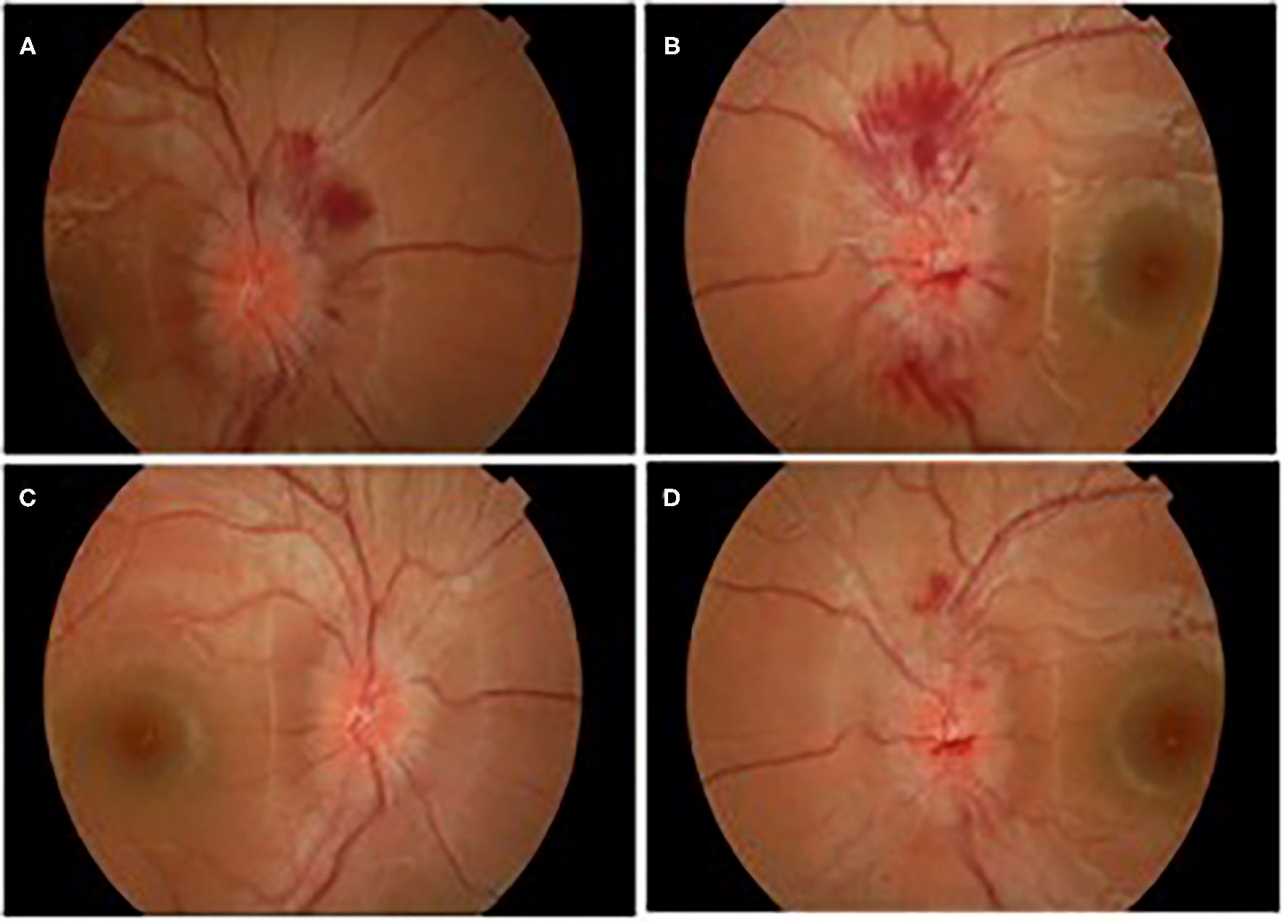
Ocular edema with flamed shaped hemorrhages OD **(A)**, OS **(B)**, and gradual improvement after treatment OD **(C)**, OS **(D)**.

A week later (18 days after IVIg treatment), severe deterioration of her physical status was noticed as she presented bilateral and relatively symmetric weakness of all limb muscles and suffered from severe headaches. Additionally, neurological evaluation revealed cranial nerve involvement with dysarthria and voice hoarseness, along with bilateral facial weakness, strabismus, weak gag reflex, and tongue paresis coupled with respiratory muscles weakness. Ocular examination showed decreased visual acuity with right esotropia and presence of bilateral optic disc edema with peripapillary flame-shaped hemorrhages (Figure 3). Although treated for hypertension, pathologic values of BP sustained (SBP/DBP: 140/90 mmHg).

**Figure 4:**
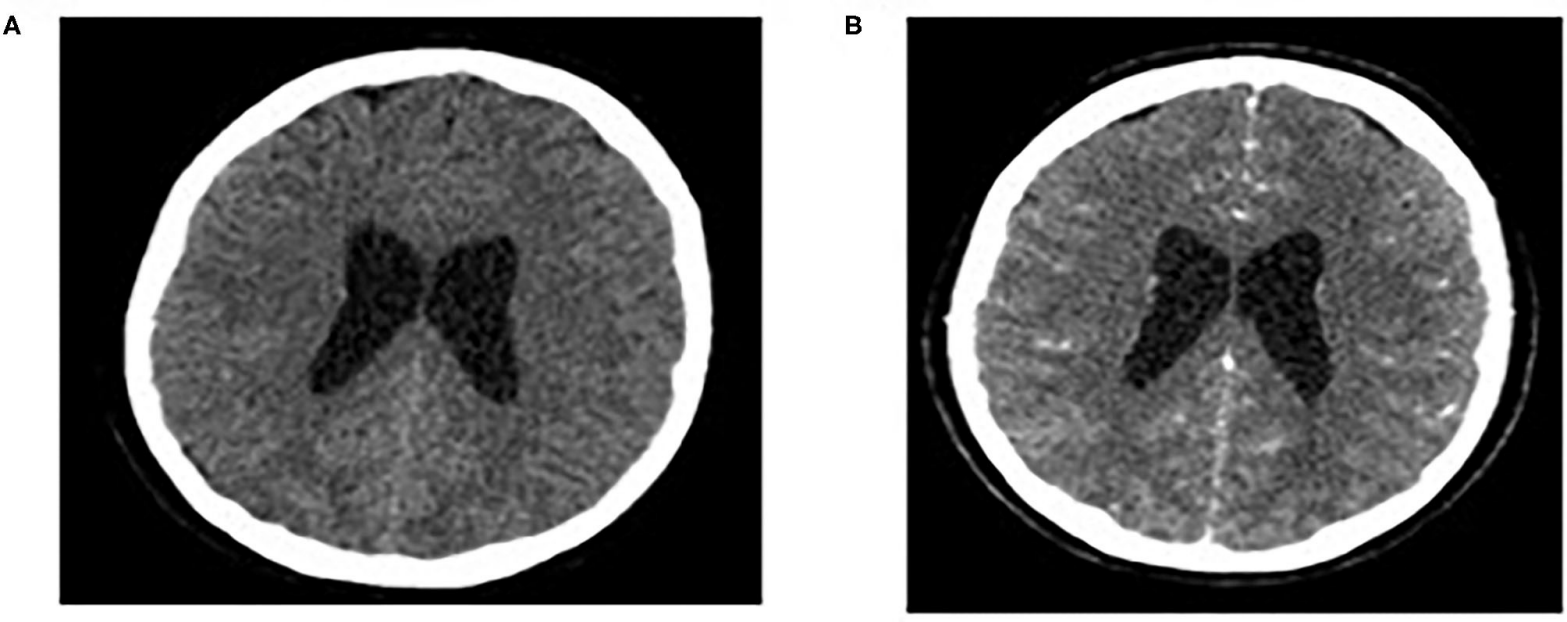
Normal findings of brain CT scan: CT brain scan without/with intravenous contrast dye administration **(A,B)**.

Brain MRI and MRA scan was not feasible due to patient's non-cooperation and sedation was considered inappropriate for her safety. Therefore, we performed urgent brain CT scan with intravenous contrast dye administration, which was unremarkable, excluding the possibility of venus thrombosis, cerebral bleeding, displacement of midline brain structures and abnormal perimedullary spaces (Figure 4). Subsequent lumbar puncture revealed a CSF pressure peaked at 46 cm H_2_O. The CSF contained 10 × 10^6^/L WBC and an increased protein level (245 mg/dL), thus a total of 30 ml CSF was removed. CSF removal was performed in consultation with neurosurgeon and continuous vitals monitoring of our patient. Due to her motor deterioration and secondary ICHP, she was promptly treated for a second time with 5 days IVIG administration (0.4 g/kg per day) and oral steroids (prednisolone: 2 mg/kg per day for 2 weeks). She was also treated with acetazolamide (250 mg four times per day for 5 months). While she was in hospital, she started having a gradual recovery, without any clinical deterioration. Continuous ophthalmologic and clinical reassessments showed a gradual and significant improvement in papilledema and visual acuity over the following 2 months. Eventually, with the aid of rehabilitation team and dietary she continued to retain function, lose weight, and remain asymptomatic over the subsequent 7 months (Figure 1).

## Discussion

GBS is the most common cause of acute flaccid paralysis in healthy infants and children (31, 32). ICHT, a pathological feature that is reported in a few scattered cases of GBS, occurs in 4–6% of children with GBS and the exact mechanism remains elusive (9–13, 19–22). In this systematic study, we present and discuss previous case reports of GBS associated with ICHT, papilledema or hydrocephalus (Table 1). We also report our current case of an obese girl with typical signs of GBS associated with cranial nerve deficits, autonomic dysfunction and increased intracranial pressure (ICP), highlighting the differences of this case compared to previous reports.

The results derived from our systemic research analysis display that the peak age incidence is 20–40 years, and there is no significant difference between sexes. To be more concise, 9/27 patients were children (average age 10 years old). The duration of the illness from onset of symptoms to complete recovery, ranged from 1 month to 2 years, with an average of about 7 months. Although weight as a parameter is not always documented, we noticed that 5/27 patients are obese, suggesting an increased possibility of overweight and obese patients to develop ICP in GBS setting. Respiratory insufficiency and mechanical support are reported in 9/27 patients (Table 1) (7, 14–16, 18, 23–29).

Diagnosis of idiopathic intracranial hypertension requires papilledema, normal neurologic exam other than cranial nerve abnormalities, normal cerebrospinal fluid (CSF) composition, elevated lumbar puncture opening pressure (≥280 mm CSF in children) and normal neuroimaging (Friedman DI, et al. Neurology 2013; 81: 1–7). Thus, in the current case, intracranial pressure is secondary to GBS leading to papilledema, headache, decreased vision acuity and worsen GBS symptoms with bilateral and relatively symmetric weakness of all limb muscles. In fact, papilledema with or without hydrocephalus is rarely reported in GBS (7, 13, 20). It appears mainly after the established limb weakness and is associated with elevated CSF protein similar to our case (12). However, it is also reported that some patients presented headache, vision disturbances, ICHT and papilledema days before GBS onset (6/27 case reports) (Table 1).

Papilledema takes time to develop and has a low sensitivity for ICHT (33). However, when present, papilledema can be a specific indicator of elevated ICP. Nowadays, there are more accurate non-invasive techniques of ICP assessment, such as sonographic measurement of the optic nerve sheath diameter (ONSD) and Transcranial Doppler (TCD) (34). The reliability of both techniques depends on the knowledge of limitations and requires experienced and skilled operators.

Although the mechanism of ICHT is not well-understood, edema of the spinal nerve rootlets seen in GBS, causes decreased proteins absorption and, thereby, elevated CSF protein levels. It is speculated that increased CSF protein concentration slows reabsorption in the arachnoid granulations resulting in raised ICP (protein absorption theory) (19, 35). The aforementioned analysis underscores a tight correlation between papilledema development and the occurrence of increased pressure of the CSF. Intriguingly, in several cases the association between development of the papilledema and variations in the CSF protein levels was quite puzzling, suggesting that certain findings seem inconsistent with protein absorption theory. Moreover, what is striking about trying to explain this theory, is that although ICHT is a rare complication of GBS, high levels of CSF protein are common enough. Additionally, there are few case reports presenting GBS patients with normal CSF protein levels. These data suggest an alternative explanation of increased ICP, implicating intrinsic cerebral edema rather than impairment of CSF reabsorption. CSF dynamic studies demonstrated high venous pressure at points of CSF outflow (8, 12, 14, 21).

The potential role of underlying immunological disturbance with activation of the classical and alternative complement pathway resulted in CSF accumulation via either impaired CSF absorption at the arachnoid villi or, alliteratively, increased production of CSF at the choroid plexus. It is suggested that GBS patients, who have a relapsing course and develop high ICP, are immunologically different from those with conventional symptoms of polyneuropathy alone (8, 12). The exact mechanism of the development of high ICP during GBS remains elusive. In the present case, we did not check complement components C4 and factor B apart from C3 that was quiet high (Table 2).

Regarding treatment, both intravenous immunoglobulin (IVIg) or plasma exchange (PLEX) are proven effective (36–38). IVIg seems to be effective in children with GBS and is preferred over PLEX because it is easier to be administrated and possibly better tolerated in young children (2, 39). Importantly, PLEX can have more adverse effects and complications in children than in adults due to citrate toxicity and higher vascular volume shifts (40). A recent study comparing PLEX and IVIg as a first line treatment for children with severe GBS requiring mechanic ventilation (MV) revealed that PLEX is superior to IVIg regarding the duration of MV but not the PICU stay or the short term neurological outcome (41). In our experience, IVIg administration is first line treatment for GBS and a choice of availability and avoidance of a prolong stay in PICU.

It has been also reported that patients with IIH or GBS have increased levels of IL-17 in both CSF and plasma (42). IVIg seems to exert its therapeutic effects on GBS by downregulating IL-17 (43). Besides IVIg and PLEX, no other procedures or drugs have been proven effective in GBS treatment (2). Although corticosteroids would be expected to be beneficial in GBS, it has been reported that they are ineffective for treating GBS and there was no significant difference between methylprednisolone—IVIg and IVIg group alone (36, 44). The lack of efficiency might be related to their adverse effects on denervated muscle or macrophage activity (45). Congruently, studies in animal models have shown that corticosteroids may reduce the recruitment of macrophages that play a crucial role for nerve regeneration, thus delay disease recovery (46).

GBS is usually a monophasic disease, but secondary deterioration after initial stabilization or improvement is possible in 5–10% of treated GBS patients (47, 48). Therefore, it is suggested that a second course of IVIg treatment especially in patients with a bad prognosis, could be effective (49, 50). Indeed, in the current case, we proceeded in a second course of IVIg treatment. Actually, the patient presented slow, but gradual improvement, upon the first IVIg administration. However, 2 weeks later, our patient suffered from a severe deterioration of her clinical status characterized by papilledema, reduced visual acuity, diplopia and persistent headaches. We suppose that our patient's overweight status was probably involved in the pathogenesis of ICP, as obesity is implicated in the development of ICP (51–54).

Although pain may be a heralding feature of GBS, it is widely documented that headache is a rare symptom. A large prospective study of pain in GBS, demonstrated 2% prevalence of headache (55). Most case reports correlate headache and GBS with posterior reversible encephalopathy syndrome [PRES], an increasingly recognized dysautonomia-related GBS complication (56). Less frequent causes of headache in GBS are secondary intracranial hypertension, cerebral venous sinus thrombosis and aseptic meningitis after IVIg administration. In the current case report, headache coincided with ICHT. Although headache could be an adverse effect of IVIg treatment, the remarkable clinical response after CSF removal argued against the possibility of post IVIg aseptic meningitis. Additionally, second lumbar puncture showed increased ICP with normal cells and elevated CSF protein levels. Moreover, urgent brain CT scan with intravenous contrast dye administration, which was unremarkable, excluded venus thrombosis.

In total, our patient was treated with two courses of IVIg, carbonic anhydrase inhibitor and steroids for facing GBS itself and intracranial hypertension. Additionally, a selective β1 receptor antagonist was used for blood hypertension. Although corticosteroids have been recommended in the past for ICHT, the long-term use should be avoided because of their side effects. Corticosteroids are indicated only on a short-term basis in patients with fulminant disease accompanied by severe papilledema and compromised visual function. Although their pathophysiological mechanism remains elusive, it is suggested that they reduce ICP primarily in vasogenic edema due to their beneficial effect on the blood vessel (57–59). In the current case, however, we used steroids empirically for short-term treatment in the setting of ICHP, acute visual loss and deteriorating clinical status. Carbonic anhydrase inhibitors can provide symptomatic relief of raised intracranial pressure by promoting the reduced CSF production at the choroid plexus. Acetazolamide is considered as the first-line medication for IIH (60). Eventually, our patient started having a gradual and significant recovery without reporting any symptomatic and functional deterioration. 7 months later the clinical evolution is excellent with complete ophthalmological and neurological recovery.

Headache, papilledema and ICHT in the setting of GBS are sparse, but potentially severe events and require further investigation. Obesity may be a predisposing factor, thus, physicians should be more aware of ICHT in an obese GBS patient. Neurological evaluation, fundus examination and low threshold for intracranial imaging should be an integral part of medical practice in case of obesity, headache, or visual changes in GBS patients. More research is needed to identify specific and potential therapeutic interventions against ICHT in GBS, in order to alleviate symptoms and improve outcome.

## Data Availability Statement

The original contributions presented in the study are included in the article/supplementary materials, further inquiries can be directed to the corresponding author/s.

## Ethics Statement

The studies involving human participants were reviewed and approved by Scientific Advisory Board University General Hospital of Heraklion, Crete, Greece. Written informed consent to participate in this study was provided by the participants' legal guardian/next of kin. Written informed consent was obtained from the participants' legal guardian/next of kin for the publication of this case report.

## Author Contributions

CD wrote the manuscript. PV supervised the project. All authors have participated to this case.

## Conflict of Interest

The authors declare that the research was conducted in the absence of any commercial or financial relationships that could be construed as a potential conflict of interest.
